# Impact of the adolescent and youth sexual and reproductive health strategy on service utilisation and health outcomes in Zimbabwe

**DOI:** 10.1371/journal.pone.0218588

**Published:** 2019-06-25

**Authors:** Lazarus Muchabaiwa, Josue Mbonigaba

**Affiliations:** 1 Economics Department, School of Accounting, Economics and Finance, University of KwaZulu-Natal, Durban, South Africa; 2 Economics Department, Bindura University of Science Education, Bindura, Zimbabwe; University of South Carolina Arnold School of Public Health, UNITED STATES

## Abstract

Poor reproductive health among youth and adolescents threatens their future health and economic wellbeing in Zimbabwe amidst a high HIV/AIDS prevalence. This study evaluates the impact of a multi-pronged adolescent sexual and reproductive health (ASRH) strategy implemented by government of Zimbabwe between 2010 and 2015 to improve ASRH in terms of the uptake of condoms and HIV testing as well as outcomes in terms of sexually transmitted infection (STI) prevalence and HIV prevalence. We combine the difference in difference and propensity score matching methods to analyse repeated Zimbabwe demographic health survey cross-sectional datasets. Young people aged 15–19 years at baseline in 2010, who were exposed for the entire five-year strategy are designated as the treatment group and young adults aged 25–29 at baseline as the control. We find that the ASRH strategy increased HIV testing amongst youth by 36.6 percent, whilst treatment of STIs also increased by 30.4 percent. We also find that the HIV prevalence trajectory was reduced by 0.7 percent. We do not find evidence of impact on condom use and STI prevalence. The findings also suggest that although HIV testing increased for all socio-economic groups that were investigated, the effect was not the same. Lastly, we do not find evidence supporting that more resources translate to better ASRH outcomes. We recommend designing future ASRH strategies in a way that differentiates service delivery for youths in HIV hotspots, rural areas and out of school. We also recommend improving the strategy’s coordination and monitoring, as well as aligning and enforcing government policies that promote sexual and reproductive health rights.

## Introduction

Young people, a collective group of adolescents aged 10–19 and youth aged 15–24, are faced with a myriad of reproductive health (RH) challenges globally [1]. These challenges consist of risks of HIV and other sexually transmitted infections (STIs), unintended pregnancies and unsafe abortions [2]. In 1994, representatives from 78 countries gathering in Cairo for the International Conference on Population and Development (ICPD) agreed on improving adolescent and youth sexual and reproductive health (ASRH) through age-appropriate health information [3]. Notwithstanding this agreement, by the end of 2015, sub-Saharan Africa still had the highest rates of new HIV infections and the highest Disability Adjusted Life Year rates amongst young people [4–6].

The ASRH challenges are currently recognised through Sustainable Development Goal (SDG) number 3, which aims to eradicate HIV infections and provide universal access to sexual and reproductive health services as well as incorporating such services into national strategies [6–8]. African countries have acknowledged the importance of ASRH and, as a result, have been implementing related strategies both at community and facility levels. These strategies have included comprehensive sexuality education (CSE), referred to as sexuality and relationship education curricula that are age-appropriate and culturally relevant [9, 10]. They have also encompassed peer education, mass media campaigns, cash transfers and youth-friendly centres- which are spaces created for young people to access ASRH health information and service [11–13], and youth-friendly services- which are accessible and appropriate services that appeal to youths in a manner that promotes equity and interactions between users and providers [14].

Studies evaluating the effectiveness of ASRH strategies to date have produced mixed results in low and middle-income countries. A descriptive review of the outcomes of these ASRH strategies at facility and community-levels found limited evidence of their effectiveness in improving ASRH outcomes amongst marginalised groups or increasing community awareness [12].

A review evaluating the impact of CSE strategies, for instance, reports its effectiveness in terms of reducing sexual risk behaviour [10, 15, 16], HIV, STIs and the incidence of unprotected sex [17, 18]. CSE was also found to delay sexual debut in African countries and improve condom use [15, 19–21]. Other evidence attribute the success of CSE to its design, theoretically and empirically linking its success to the right age-targeting [6] and consideration of gender power differences [9]. Other interventions imparting knowledge such as media campaigns and life skills training have been found to reduce the prevalence of STIs and multiple sexual partners and to increase condom use, abstinence and health service utilisation [22].

Other interventions that have proven to be successful are those that have targeted poverty through subsidies and cash transfers, as a way of deterring young people from risky RH practices in pursuit of income [23]. For instance, cash transfers were found to reduce pregnancy, early sexual debut (sex before age 18) and early marriage amongst female adolescents from poor backgrounds in Kenya and South Africa [23–25]. Cash transfers also reduced the prevalence of STIs among adolescents in school albeit not in those already out of school in Malawi [26]. In Kenya, the effectiveness of subsidies in reducing pregnancy and STIs was established when combined with CSE [27].

Whilst these studies have enriched our understanding of how specific ASRH interventions influence service utilisation and health outcomes, they have a particular drawback. Evaluating interventions in isolation, in limited number of combinations and in selected settings assumes an artificial and abstract environment and ignores many complex interrelated factors operating at different levels to influence ASRH outcomes in a natural socio-political setting [5]. Our study addresses this drawback by evaluating multiple complementary interventions scaled up at the national level in Zimbabwe in a non-abstract setting. This is the first such study, to the best of our knowledge, evaluating the impact of the ASRH strategy in Zimbabwe. We also add to the literature, subgroup analysis by gender, wealth and place of residence to establish equity, which is missing from most ASRH evaluations [12].

## The Zimbabwe ASRH strategy

To improve ASRH outcomes, Zimbabwe implemented the ICPD action plan through various policies including the National Reproductive Health Policy, Zimbabwe National HIV and AIDS Strategic Plan, National Health Strategy and the Educational Policy. Poor ASRH outcomes such as high-risk sexual activity involving paid sex or sex with an older partner, an increase in STIs, and low uptake of HIV testing as well as barriers to access youth-friendly services were evident in the 2005/06 Zimbabwe Demographic Health Survey (ZDHS) [28]. In response to these challenges, the government developed its first ASRH strategy for implementation between 2010 and 2015.

Amongst the barriers identified as aggravating ASRH outcomes include the lack of provision of comprehensive social and behaviour change communication (SBCC) materials; lack of life skills education, inadequate ASRH outreach services, prohibitive transport costs to referral health facilities. Only youth above 16 years of age were allowed voluntary HIV counselling and testing. Awareness of ASRH issues and skills to deal with them were low amongst health staff. Whilst there was no properly defined ASRH package across the Zimbabwean health system, commodities required for ASRH program implementation were in short supply.

The first five-year ASRH strategy implemented in 2010 aimed to integrate socioeconomic, psychological and physical factors through a multi-sectoral and participatory method involving adolescent and youth at all levels of programming in addressing barriers pointed out above. It departed from previous non ASRH specific strategies, which were curative by design to focus more on preventative measures for sexually active young people and address barriers to service utilization.

The goal of the strategy under evaluation in this paper was to improve the sexual reproductive health of young people in Zimbabwe. One of the strategy’s objectives were to encourage youth to practise safe sexual and reproductive health habits such as delaying or having protected sex, avoiding multiple sexual partners, and periodic HIV testing. Other objectives of the strategy included expanding access, availability and use of youth-friendly ASRH services, facilitating a policy environment supportive of youth-friendly ASRH services and strengthening ASRH programme coordination and partnerships. The five year strategy focused on changing risky sexual behaviour among young people, imparting life skills, providing youth-friendly services and improving policy, advocacy and coordination [29].

Three main points of contact with the eligible beneficiaries were identified as the community, health facility and school. Community youth friendly centres were established to offer sexuality education, counselling, recreational activities and condoms in the community. Health facilities availed a room and other available space for a youth-friendly corner that provided voluntary testing and counselling, condoms, family planning material and other related services. Life skills, CSE and counselling were also initiated in schools through teachers and peer educators.

Eligibility into the program interventions was based on age ranging from 10 to 24 years. This means only youth and adolescents aged between 10 and 24 years between 2010 and 2015 could access youth-friendly corners, youth-friendly services and were a target for youth-friendly awareness activities through the hospital, school and community. Age groups 25 and above were ineligible for the ASRH program but were exposed to the business-as-usual approach of accessing health services through the normal primary health facilities. For purposes of this study, adolescents are aged between 10 and 19 years whilst youth are aged between 15 and 24 years in line with the WHO and the Zimbabwean ASRH strategy definitions [28]. In addition to that, there is a clear age overlap between the two groups such that the terms “adolescents” and “youth” are used interchangeably though out the study.

**Fig 1:**
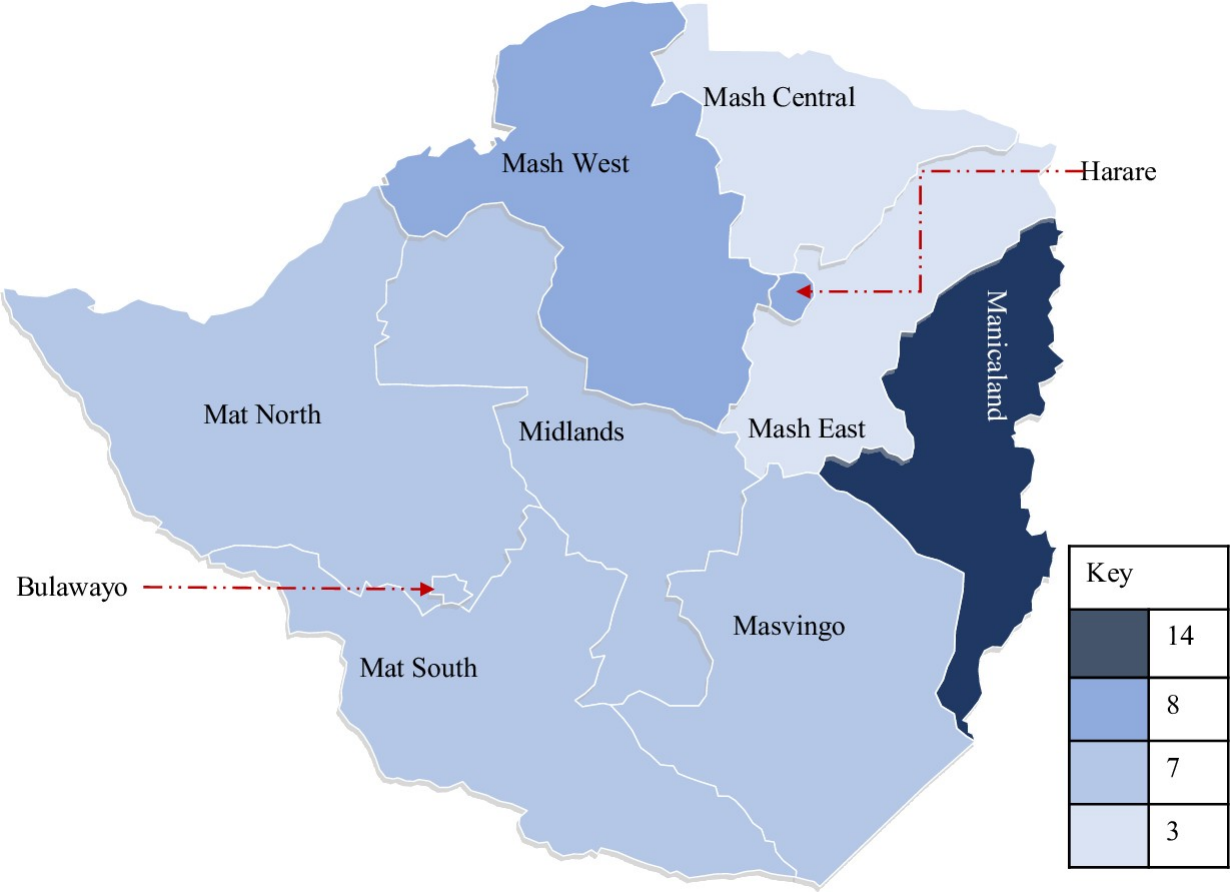
Programs per province. 14 programs were implemented in Manicaland province which is higher than any other province. Eight programs were implemented in Harare and Mashonaland West whilst 7 programs were implemented in Bulawayo, Masvingo, Matabeleland North, Matabeleland South and Midlands. Mashonaland East and Mashonaland Central had only 3 programs implemented which was the least [29].

United Nations agencies, the government, international development agencies, international and local non-governmental organisations (NGOs) funded various ASRH programs between 2011 and 2015. Whilst local NGOs and the Ministry of Health and Child Care (MoHCC) implemented them, Zimbabwe National Family Planning Council (ZNFPC), the National AIDS Council (NAC) and MoHCC were coordinating. The programs, however, were not implemented uniformly across the country as shown in Table 1 and Fig 1.

**Table 1:** Distribution of ASRH program by province.

Province	Programs implemented^#^	Population density^+^	Youth population per program^+^	poorest quintile*	Never attended school*	Total fertility rate^&^	Teenage Pregnancy^&^	HIV prevalence 15–24^&^
National Level	17	13 061 239				4.1	23.5	5.5
Bulawayo	7	5%	31,565	0	1.9	2.8	11	5.9
Harare	8	16.3%	84,333	0	1.6	3.1	20.3	5.9
Manicaland	14	13.4%	41,188	16.0	5.1	4.8	27	3.4
Mashonaland Central	3	8.8%	125,151	23.8	9.6	4.5	30.3	5.2
Mashonaland East	3	10.3%	145,255	11.5	5.2	4.5	25.1	7
Mashonaland West	8	11.5%	61,966	23.7	6.8	4.5	23.6	4.4
Masvingo	7	11.4%	69,319	23.2	6.6	4.7	23.3	4.7
Matabeleland North	7	5.7%	36,595	69.5	8.8	4.1	31.1	8.5
Matabeleland South	7	5.2%	19,136	33.1	5.5	4.2	23.1	9.2
Midlands	7	12.4%	78,087	25.5	5.3	4.2	23	4.9

Sources

^#^ [29]

^+^ [30]

* [31]

^&^ [32]

Manicaland province had the highest number of programs implemented, whilst Mashonaland East and Mashonaland Central provinces had the least as shown in column 2 of Table 1 and Fig 1. A number of reasons might explain why Manicaland province received the most attention. First, the province has the highest adolescent population in the country [33]. Secondly, sexual activity amongst female teenagers remained high in contrast to declining patterns observed amongst their male counterparts [34]. Thirdly, the region has the highest fertility rate at 4.8 against a national average of 4.1 and the highest teenage pregnancy rate at 27 percent against 23.5 percent national average [35]. Lastly, a cohort study conducted prior to the ASRH strategy showed HIV prevalence amongst teenagers in the region rising from 1.2 percent to 2.23 percent [36]. We exploit this uneven distribution of ASRH programs in Zimbabwe to assess whether program intensity resulted in better ASRH outcomes. Due to differences in population across the provinces, we also consider the number of programs per population in each province. Column 4 of Table 1 shows the inverse of this statistic presented as youth population per program. Matabeleland South province, for example, has the least population density. Its youth population per program at 19,136 is the lowest compared to 145,255 in Mashonaland East.

## Hypothesis

In an attempt to analyse the impact of the Zimbabwean ASRH strategy, this study first evaluates the effect of the strategy on condom utilisation, STI treatment, HIV testing, HIV prevalence and STI prevalence. It then disaggregates the effect by gender, household wealth and residential location. Lastly, the study evaluates whether policy intensity, as measured by the number of programs implemented per province as well as the number of programs per population in a province, would lead to superior ASRH outcomes. The null hypothesis is that that the multi-pronged Zimbabwean ASRH strategy had no bearing on ASRH service utilisation and outcomes.

## Methods

### Empirical framework

Randomisation is widely accepted as a gold standard for programme impact evaluation [37, 38]. Due to social policies that are not implemented with randomisation in mind, economists estimate their effects using adjusted regressions, matching techniques, regression discontinuity, instrumental variables, as well as difference in difference (DID) techniques. A combination of these methods with propensity score matching has been shown to yield estimates close to randomised experiments [39, 40].

Our study combines propensity score matching and DID methods. The DID method is appropriate for the current study due to its before and after design. It is designed for panel and repeated cross-section data [41–43] and accounts better for time invariant unobserved heterogeneity [41]. Propensity score matching allows for the construction of a counterfactual and reduced selection bias [44, 45]. Using propensity score matching to complement DID facilitates balanced matching of treated and control observations [41].

In a study closely related to our approach, Stuart et al. develop and test a model that combines propensity score matching and the DID approach to policy evaluation [43]. This approach involves the construction of propensity scores from four groups: treatment group at baseline, treatment group post-policy, control group at baseline and control group post-policy. The researchers implement this approach for repeated cross-sectional data on an innovative payment and delivery system on out-of-pocket health expenditures. They find that the new payment and delivery system did not result in increased out-of-pocket expenditures. The drawback, however, associated with this approach is increased standard errors. This is a consequence of the bias-variance trade-off, such that obtaining less biased impact estimates is associated with a cost of higher variance [43]. The current study makes use of the *diff* estimand developed by Villa [41], which combines the DID approach and kernel-based propensity scores in Stata statistical software.

### Econometric approach

Our econometric specification is guided by the fact that we are seeking to establish the difference across two groups over time which is attributable to the ASRH strategy. The effect of the ASRH strategy is obtained from estimating the equation:

$$y_{i}=\alpha_{0}+\beta_{0}T_{i}+\beta_{1}t_{i}+\delta_{1}T_{i}t_{i}+\beta_{ij}\sum X_{ij}+\varepsilon_{i}$$ (1)

where *y*_*i*_ is health indicator for person *i*

*t*_*i*_ is a dummy variable for time taking the value zero for 2010 observations and one for 2015 observations.

*T*_*i*_ is a dummy taking the value one for the treatment group and zero otherwise.

#### Treatment group

Eligibility into the program was based on age. The ASRH strategy was implemented across the whole country and targeted young people aged 10–24 years. The study selected young people aged 15–19 years old in 2010 as the baseline treatment group as it benefited over the entire five-years of the strategy’s implementation. By the end of the strategy implementation in 2015, the cohort was now aged 20–24, which becomes the post-strategy treatment group. Although targeted by the strategy, young people aged 10–14 years were excluded from the analysis because there is no secondary data on sexual and reproductive health information available for Zimbabwe.

#### Control group

The 25–29 years age group in 2010 never benefitted in the strategy implementation period. Instead, it was exposed to the business-as-usual sexual and reproductive health approach and becomes the baseline control group. By 2015, this cohort was now aged 30–35, which also was not exposed to the ASRH programme and thus becomes the post-strategy control group.

The study further analyses whether higher program intensity brought about better results. In this sub-analysis, the eligibility criterion was region of residence whereby ASRH beneficiaries from Manicaland province which received the highest number of interventions were considered as the treatment group whilst those from Mashonaland East and Central provinces which received the least programs were considered as the control group. Youth aged 15–19 resident in Manicaland province which had 14 ASRH programs were selected as the treatment group at baseline using the 2010 dataset. At follow up, the same group from the same province were aged 20–24 in the 2015 dataset and were thus considered as the treated group at follow up. Youth of the same ages from provinces with the least number of ASRH programs (Mashonaland Central and Mashonaland East with 3 programs) were considered as the control groups. Following the same reasoning, we assess the differences between Matabeleland South province which has the lowest youth population per program at 19,136 and Mashonaland East which has 145,255.

*X*_*i*_ represents covariates identified from the literature, these being residence, wealth status, gender and marital status. The difference post exposure (*t* = 1) is obtained by subtracting health outcome indicators of the control (*T* = 0) from those of the treated (*T* = 1) post strategy:

$$(E[y_{i}|t_{i}=1;T_{i}=1]=\alpha_{0}+\beta_{0}+\beta_{1}+\delta_{1})-(E[y_{i}|t_{i}=1;T_{i}=0]=\alpha_{0}+\beta_{0})=\beta_{1}+\delta_{1}$$ (2)

The difference at baseline (*t* = 0) is obtained by subtracting health outcome indicators of the control (*T* = 0) from those of the treated (*T* = 1) before the strategy:

$$(E[y_{i}|t_{i}=0;T_{i}=1]=\alpha_{0}+\beta_{1})-(E[y_{i}|t_{i}=0;T_{i}=0]=\alpha_{0})=\beta_{1}$$ (3)

The DID estimator is obtained by subtracting the average in Eq 3 from Eq 2 [41,42]:

$$(\beta_{1}+\delta_{1})-\beta_{1}=\delta_{1}$$ (4)

In this respect, the DID estimate is the change in the difference in group (treatment) outcomes across time.

### Data

The data consist of repeated cross-sectional ZDHS datasets collected before ASRH implementation in 2010 and at the end of the strategy period 2015. The design of the survey, which has been conducted every five years since 1988, is independent from the design of the ASRH strategy and, thus, eligibility to treatment and control groups makes the study a quasi-natural experiment. ZDHS datasets are large nationally representative samples collected every five years, which allows for policy analysis on pooled cross-sectional data. No other survey carried out at the national level collects data on health indicators as comprehensive as the ZDHS. In addition to that, the survey design is subject to international standards as it is implemented in over 100 countries globally.

### Dealing with estimation issues

#### Selection bias across time and across groups

Changes in group composition and time trends are a source of selection bias [43, 46] when applying the DID method. Since the study uses repeated cross-sectional data, there is no identifier in the datasets linking individuals sampled in the second period to the first period. The potential for group composition changes, a common feature of repeated cross-sectional surveys [43] necessitates the control for selection bias. Propensity scores are used to address selection bias.

#### Propensity score weighting

The propensity scoring approach, proposed by Rosenbaum and Rubin, is used to minimise selection bias from changes in group composition [45]. Kernel propensity scores are used to make the post-ASRH strategy groups (treated and control) similar to the pre-ASRH strategy groups (treated and control) using observed baseline characteristics. Data cleaning, combination of datasets and data analysis were conducted in Stata 13 statistical software.

## Results

### Socioeconomic variables

Table 2 provides characteristics of control and treatment groups and statistical significance of their differences. It shows that chi-square tests of independence for categorical variables are all significant, suggesting that the data are not balanced on the socioeconomic variables across the four groups. The data need to be balanced to reduce selection bias. The differences in age and wealth index as continuous variables were diagnosed using one-way ANOVA, which showed significant differences. The age difference is expected due to deliberate selection of these age groups for the strategy evaluation.

**Table 2:** Socioeconomic characteristics by group.

Group	1	2	3	4	p-value of differences
Year	2010	2010	2015	2015	
Treatment	Treatment	Control	Treatment	Control	
**Variable**					
**Age (Mean)**	17	27	22	32	0.00
**Wealth Index (Mean)**	37.68	47.97	36.07	43.28	0.00
**Gender %**					
Female	52.10	56.31	61.11	60.03	0.00
Male	47.90	43.69	38.89	39.97	
**Religion %**					
Apostolic Christians	32.5	32.438	32.82	36.11	0.03
Other	67.5	67.562	67.18	63.89	
**Education %**					
None	0.42	24.61	74.49	0.49	0.00
Primary	0.39	19.77	72.02	7.81	
Secondary	1.01	25.23	67.36	6.40	
Higher	0.59	23.18	65.25	10.98	
**Residence %**					
Rural	70.77	53.55	65.49	54.96	0.00
Urban	29.23	46.45	34.51	45.04	
**Observations**					10,247

Significance of differences in categorical variables was established using chi-square test while difference in continuous variables was done using the ANOVA test. Data for age and wealth index (continuous variables) are in real numbers while all other data are percentages. Proceeding to the DID analysis with such unbalanced data leads to the selection bias problem highlighted earlier. Propensity scores are used to balance data for socioeconomic variables. Only a few variables, however, can be used for balancing in order to make the balancing procedure feasible [44].

### Difference in difference estimation results

Tables 3–6 show results of DID analysis for six models estimating the impact measures of ASRH strategies with respect to condom use, STI prevalence, STI treatment, HIV testing and HIV prevalence by comparing results of the treatment group relative to the control group. The “Before” panel shows estimates of the differences in the outcome indicator − change in condom use for instance − between the treatment and control groups before the 2010 ASRH strategy. The “After” panel shows estimates of the differences in the outcome indicator between the treatment and control groups at the end 2015. The last row labelled Diff-in-Diff shows the impact estimated, which is equal to the difference in the “After” panel estimates minus the difference in the “Before” panel estimates.

**Table 3:** National level strategy impact.

	(1)	(2)	(3)	(4)	(5)
VARIABLES	Condom use	STI Prevalence	STI treatment	HIV Testing	HIV Prevalence
*Before*					
Control	0.733	0.028	0.528	0.562	0.153
Treated	0.683	0.003	0.282	0.186	0.038
Diff (T-C)	-0.050	-0.025	-0.246***	-0.376***	-0.114***
	(0.088)	(0.007)	(0.080)	(0.022)	(0.018)
*After*					
Control	0.713	0.026	0.459	0.780	0.189
Treated	0.760	0.022	0.517	0.769	0.067
Diff (T-C)	0.047	-0.004	0.058	-0.011	-0.121***
	(0.108)	(0.013)	(0.111)	(0.036)	(0.027)
*Diff-in-Diff*	0.097	0.021	0.304**	0.366***	-0.007***
	(0.139)	(0.015)	(0.137)	(0.042)	(0.032)

Standard errors in parentheses

*** p<0.01

** p<0.05

* p<0.1

**Table 4:** Difference-in-differences estimation results Manicaland vs Mashonaland Central Province.

	(1)	(2)	(3)	(4)	(5)
VARIABLES	Condom use	STI Prevalence	STI treatment	HIV Testing	HIV Prevalence
*Before*					
Control	0.500	0.006	0.419	0.185	0.022
Treated	0.500	0.004	0.345	0.190	0.024
Diff (T-C)	0.000	-0.003	- 0.074	0.005	0.001
	(0.526)	(0.005)	(0.227)	(0.028)	(0.011)
*After*					
Control	0.696	0.017	0.617	0.762	0.061
Treated	0.880	0.051	0.700	0.784	0.039
Diff (T-C)	0.184	0.034	0.083	0.022	-0.022
	(0.153)	(0.016)	(0.175)	(0.039)	(0.021)
*Diff-in-Diff*	0.184 (0.548)	0.037** (0.017)	0.157 (0.287)	0.018 (0.048)	-0.023 (0.024)

Standard errors in parentheses

*** p<0.01

** p<0.05

* p<0.1

**Table 5:** Difference-in-differences estimation results: Manicaland vs Mashonaland East Province.

		(1)		(2)		(3)		(4)		(5)	
VARIABLES		Condom use		STI Prevalence		STI treatment		HIV Testing		HIV Prevalence	
*Before*											
Control		0.748		0.003		0.000		0.189		0.052	
Treated		0.491		0.005		0.250		0.201		0.025	
Diff (T-C)		-0.257		0.002		0.250		0.012		-0.028	
		(0.341)		(0.004)		(0.227)		(0.030)		(0.015)	
*After*											
Control		0.844		0.031		0.575		0.793		0.065	
Treated		0.913		0.051		0.750		0.784		0.039	
Diff (T-C)		0.069		0.020		0.175		-0.008		-0.026	
		(0.108)		(0.019)		(0.213)		(0.039)		(0.021)	
*Diff-in-Diff*		0.326		0.018		-0.075		-0.020		0.002	
		(0.358)		(0.019)		(0.311)		(0.049)		(0.026)	

Standard errors in parentheses

*** p<0.01

** p<0.05

* p<0.1

**Table 6:** Difference-in-differences estimation results: Matabeleland South vs Mashonaland East Province.

		(1)		(2)		(3)		(4)		(5)	
VARIABLES		Condom use		STI Prevalence		STI treatment		HIV Testing		HIV Prevalence	
*Before*											
Control		0.922		0.000		0.000		0.169		0.052	
Treated		0.712		0.002		0.334		0.175		0.042	
Diff (T-C)		-0.210		0.002		0.334		0.006		-0.010	
		(0.202)		(0.002)		(0.293)		(0.030)		(0.017)	
*After*											
Control		0.860		0.025		0.562		0.764		0.069	
Treated		0.640		0.023		0.636		0.837		0.152	
Diff (T-C)		-0.220*		-0.002		0.074		0.073*		0.083***	
		(0.127)		(0.014)		(0.223)		(0.042)		(0.030)	
*Diff-in-Diff*		-0.010		-0.004		-0.260		0.067*		0.093***	
		(0.238)		(0.014)		(0.368)		(0.052)		(0.034)	

Standard errors in parentheses

*** p<0.01

** p<0.05

* p<0.1

In order to effectively increase demand for ASRH services, implementers also involved parents as community gate keepers [28], which raised the need to adjust our control group such that it did not include parents of targeted youth. We thus dropped households that had siblings targeted by the strategy to control for spill over effects in the control group for the 2015 dataset.

#### Condom use

The ZDHS survey asked respondents about condom use during last sexual encounter with most recent partner as well as the number of sexual partners in the 12 months preceding the survey. We created a variable combining these two aspects to investigate condom use by individuals with more than one sexual partner in the last 12 months. As shown in Table 3, condom utilisation by individuals in the treatment group who were sexually involved with at least two partners in the past 12 months was lower than the control group by 5 percent at baseline. After the strategy’s implementation, utilisation in the treatment group was higher by 4.7 percent. The overall effect of the ASRH strategy was a rise in condom utilisation in the treatment group by 9.7 percent (diff-in diff row) albeit not statistically significant. The evidence is insufficient to conclude that there was any significant improvement in condom utilisation by the targeted group.

#### STI prevalence

At baseline, STI prevalence was 0.3 percent for the treatment group compared to 2.8 percent for the control group and a difference of 2.5 percent. Post-strategy, it increased to 2.2 percent and 2.6 percent for the treatment and control groups respectively. The overall effect of the strategy is not statistically significant and thus, insufficient evidence to conclude that the ASRH strategy had any bearing on STI prevalence.

#### STI treatment

At baseline, only 28.2 percent of STI infected young people sought treatment compared to 52.8 percent in the control group. Post-strategy, the treatment of STIs amongst young people increased to 51.7 percent but declined for the control group to 45.9 percent. The overall effect of the strategy was an increase in STI treatment of 30.4 percent statistically significant at the 5 percent level.

#### HIV testing

The proportion of the targeted group who had ever had an HIV test before the strategy was 18.6 percent, which was lower than the control group’s 56.2 percent. After the implementation of the ASRH program, the proportion of the young people ever tested had increased to 76.9 percent whilst the control group increased to 78 percent. The overall change in the proportion ever tested for HIV attributable to the ASRH strategy on the treatment group was an increase in the proportion tested of 36.6 percent, which is statistically significant at the 1 percent level.

#### HIV prevalence

The prevalence of HIV for the treatment group was 3.8 percent at baseline. It was 11.4 percent lower than the control group with 15.3 percent prevalence, but the gap was statistically insignificant. After the implementation of the strategy, the gap widened to 12.1 percent significant at the 1 percent level where the prevalence had increased to 6.7 percent for the treatment group and 18.9 percent for the control group. The difference in difference estimand suggests that although HIV prevalence increased in the targeted group, the ASRH strategy managed to lower its trajectory by 0.7 percent significant at the 1 percent level.

#### Subgroup analysis

The DID analysis was further conducted by subgroups of gender (see S1 Table, household wealth (S2 Table) and residential location (S3 Table). The analysis revealed that the ASRH program increased HIV testing for both males and females significantly at the 1 percent level and that the rise was more marked in females. HIV testing also went up significantly across wealth status and place of residence. The increase in HIV testing was higher for urban residents than for their rural counterparts and higher for treatment group members from rich households all significant at the 1 percent level. HIV prevalence reduction was significant amongst urban youth but no significant change amongst their rural counterparts. STI prevalence increased significantly amongst rural youth without significant change amongst urban youths.

The strategy increased the treatment of STIs for both males and females. STI treatment also increased amongst young people in rural areas but that coincides with a rise in STI prevalence in the same group as well. No effect was noted on condom use across all subgroups.

### Program intensity

A question that is worth answering is whether higher program intensity brought about better results. To answer this question, the impact of ASRH was analysed by using the province with the highest number of ASRH programs (Manicaland with 14) as the treatment group and provinces with lowest number of ASRH as control groups (Mashonaland Central and Mashonaland East with 3 programs). Tables 4 and 5 present the effects of higher program intensity on ASRH outcomes. In Table 4, results of Manicaland province are compared to Mashonaland Central while in Table 5 they are compared to results of Mashonaland East.

As Table 4 shows, condom use in Manicaland province and Mashonaland Central were similar before the strategy. After the ASHR strategy, condom utilisation rose in both provinces but was higher in Manicaland by 18.4 percent in line with expectations of more programs implemented. The overall impact, however, was statistically insignificant and thus, we cannot conclude that higher program intensity translated to improvement in condom utilisation.

Whilst STI prevalence increased by 3.7% in the treatment group, significant at the 5 percent level, the corresponding increase in STI treatment by 15.7 percent is not significant. HIV testing also increased by 1.8 percent for Manicaland but it is statistically insignificant. The rise in HIV prevalence in Manicaland province was lower than that of Mashonaland Central giving a DID estimate of 2.3 percent, which is not statistically significant. We thus do not find evidence of better outcomes with higher program intensity for condom use, STI treatment, HIV testing and HIV prevalence.

In Table 5, condom use increased by 32.6 percent, whilst STI prevalence and HIV prevalence went up by 1.8 percent and 0.2 percent respectively. Treatment in STIs and HIV testing also went down in Manicaland relative to Mashonaland East province. None of these changes is statistically significant, thus, we do not find evidence of any effects of higher intensity in the ASRH strategy implementation.

An alternative way to assess intensity was to consider the number of programs per population in a province. Matabeleland South province had the least youth population per program in contrast to Mashonaland East. Since programs relate to resources committed towards improvement of ASRH indicators, this statistic suggests that resources in Mashonaland East were thinly spread on a higher population in contrast to Matabeleland South province. We would thus expect superior outcomes for Matabeleland province. Table 6 shows DID results of Matabeleland South as the treatment group and Mashonaland East as the control. The results suggest that Matabeleland South had a superior outcome only for HIV testing which was 6.7% higher than Mashonaland East which is statistically significant at the 10 percent level. Matabeleland South province had a higher HIV prevalence relative to Mashonaland East. This finding contrasts with expectations as well as the insignificant impact on condom utilization, STI prevalence and treatment.

## Discussion

Poor sexual and reproductive health among young people threatens their future, especially in countries with high HIV prevalence [47–50]. To preserve the well-being of these young people, effective interventions need to be ascertained or improved through evaluation and monitoring.

The undertaking of our impact evaluation study was premised on the expectation that the implementation of multiple complementary interventions would result in synergies of the interventions and in better outcomes. In addition to that, we expected that more resources employed in some provinces would lead to much better outcomes. The findings suggest that the strategy had significant effect on increasing the treatment of STIs, increasing HIV testing and reducing the HIV prevalence trajectory. Subgroup analysis showed that the ASRH program increased the uptake of HIV testing regardless of gender, household wealth and residential location. Even more encouraging was the finding that the rise in HIV testing was higher amongst females, who are normally left behind. Our evidence, however, does not support any impact on the use of condoms nor reduction of STI prevalence. Furthermore, the study found mixed evidence on the effect of higher program intensity.

The finding of HIV prevalence trajectory falling is in line with declining national level HIV prevalence from a high of 25.6 percent at its peak in 1997 to 15.2 percent in 2010 and now to 13.4 percent in 2015 [32, 33, 51, 52]. Declining HIV prevalence can be brought about by HIV-infected people dying or decline in new infections, but recent scientific evidence suggests the latter [53–57]. The ZIMPHIA report of 2016 shows that HIV incidence fell from 0.88 percent to 0.5 percent in 2015, which is attributed to progress in controlling the epidemic [33]. Hargrove et al [53] relate the declining pattern to emigration, reduction in risky sexual behaviour, scaling up of voluntary HIV counselling and testing as well as prevention of mother to child transmission and increased knowledge about HIV and AIDS. Increase in knowledge is also supported by the 2015 ZDHS report which shows that knowledge of HIV prevention methods increased by 4 percent from 78 percent in 2010 [51]. Halperin et al [54] establish a positive association between the declining trends and reduction in extramarital, commercial, and casual sex relations; along with mass media and church based prevention activities in Zimbabwe. Gregson et al [55] find the decline in HIV prevalence associated with the scaling up of ART in Zimbabwe, declining risky sexual behaviours, declining multiple sexual partners and reduced involvement with commercial sex workers.

Our study found increasing HIV testing and a lower HIV prevalence trajectory occurring with no significant change in condom use. This leads us to conclude that other prevention approaches identified in literature had a dominant effect on reducing new infections. These include risk reducing behaviour namely extramarital, commercial, and casual sex relations complemented by CSE, mass media, scale up of voluntary counselling and testing and other church based prevention activities [54, 55]. Scientific evidence reviewed earlier, also indicates that CSE and media campaigns have a positive effect in reducing sexual risk behaviour [10, 15, 16] and HIV and STIs [17, 18].

We attribute the lack of impact on condom utilisation to legal barriers in the distribution of condoms and contraceptives to school going youth in Zimbabwe. Despite evidence that 41 percent of female youths are sexually active by the age of 18 [51], the government does not allow distribution of condoms in schools [58]. This does not only contrast with the ASRH strategy, but it also works against the spirit of promoting safe sex, which as evidenced by this study, affects condom uptake beyond school-going years. Chandra-Mouli et al. [13] refers to such implementation as a piecemeal approach to ASRH. Such barriers to distribution of such a critical commodity can reverse gains of CSE pointed out in the literature review instead of complementing them. This could also have contributed to the lack of progress in reducing STI prevalence also established in our results.

STI treatment is important as it helps in managing STI incidence, STI prevalence [57] and HIV infection [59]. Under the ASRH strategy, STI patients were also offered HIV testing and counselling to facilitate containment of HIV incidence as well as encouraging treatment of the sexual partner. Our finding of STI treatment improving and HIV trajectory going down suggests a success story of the STI management approach adopted and resonates well with findings elsewhere in Africa [60]. This finding entails the need for more resources for the current STI management approach to consolidate gains as well as stocking hard to reach health facilities with medicines and HIV testing kits.

The lack of impact by high intensity implementation of the ASRH strategy comes against the study expectations. We expected better outcomes in Manicaland, which had more ASRH programs implemented in comparisons to other provinces. The finding suggests poor coordination of ASRH programs by implementers as highlighted by Blum et al and Marimo et al [29, 61]. Marimo et al attribute the lack of impact of the ASRH strategy to poor implementation [61], which was also highlighted by Michielsen et al for interventions covered across 28 studies in Africa [15]. This evidence suggests more resources do not necessarily lead to better outcomes but that there is need for better packaging of the combined strategies, which can be done by looking at the design of each component, the distributions and their interactions.

The unexpected increase of HIV prevalence in Matabeleland South has been observed in national reports before [33, 62–64]. HIV hotspots have been identified in artisanal mining areas and border towns in the province [63]. We suggest the use of pre and post exposure prophylaxis with high adherence monitoring which have been found more effective in recent literature for such high-risk groups because they do not apply protective measures consistently [65–68].

The results obtained in this study have some precedent in the literature. Whilst the literature reviewed arrived at mixed evidence with respect to ASRH strategies, this study indicated that the ASRH strategy was successful in combating the HIV epidemic through increasing testing, reducing prevalence trajectory and increasing treatment of STIs. Such success in the literature has been attributed to right age-targeting [6] and targeting of specific marginalised communities [69]. Furthermore, this study does not find major discrepancies across socioeconomic groups; however, it suggests that there can be improvements in young people’s sexual and reproductive health by revising government policy on condom distribution.

The Lancet Commission on adolescent health and well-being and other researchers have suggested implementation of multi-interventions to complement each other to improve ASRH outcomes in pursuit of SDG3 [5, 13, 70, 71]. Our results on policy intensity suggest the need of going beyond multi-pronged interventions to consider how to synergise them at the design and implementation stages. The implementation of HIV prevention strategies involved different program implementers each championing a particular intervention or set of interventions [29]. There was no framework to guide coordination or monitoring and evaluation of the various players [29, 61]. We suggest development of a coordination framework to ensure that the efforts of the different implementers complement each other and to avoid duplication of roles which wastes resources which could be useful elsewhere. Such a framework has to guide implementers as they design their workplans. In addition, there is need to align government policies from the status quo where on one hand, the ASRH strategy promotes uptake of contraceptives whilst on the other, the government policy prohibits distribution of condoms in schools [58]. Without such changes, some components might in fact play a deleterious role, with the likelihood of cancelling the effect of the overall combination.

In light of the evidence in this study and previous studies we recommend better design of interventions and alignment of the strategy to laws to reduce barriers of the strategy’s implementation. We also recommend better coordination of the various implementers to facilitate synergies to ensure better results of the strategy. To reduce STIs and HIV prevalence in identified HIV hotspots, we recommend scaling up pre and post exposure prophylaxis together with increased adherence monitoring. HIV hotspots like mining areas are also characterised by sexual violence against women which hinders adoption of safe sexual practices [72]. We thus suggest that law enforcement agents enforce human rights in general and sexual and reproductive health rights in particular in the HIV hotspots. To reduce STI prevalence particularly in rural areas, the ASRH program design can be improved by differentiating the approaches used to deliver services for youths in school relative to those out of school, to reach at risk populations in HIV hotspots relative to those in less risky locations, as well as those in urban versus those in rural areas. Furthermore, delivering ASRH services through youth friendly centres needs more monitoring to avoid them being dominated by male youths at the expense of females as found by Blum et al [29], particularly in rural areas which are more patriarchal societies.

Lastly, we recommend improvement of CSE content to address gender and power to improve outcomes amongst females, ground it on proper theories, implement it properly and target the right groups at the right time. Interventions addressing gender and power have been found five times more effective elsewhere [9]. Mwale and Muula find evidence of better outcomes for CSE programs with proper theoretical grounding, implemented properly and targeting the right groups at the right time in 17 studies carried out in Africa [6].

Limitations of this study are worth mentioning. The ASRH program was implemented across the whole country, implying that there was no perfect control group to compare to the targeted group. We ended up using the age group closest to the treatment group with only a difference of five years between them, which was not the target of the ASRH strategy. Duflo uses a similar identification strategy when studying a social experiment in Indonesia [73]. There is high chance that the post strategy control group could have been contaminated due to the presence of ASRH beneficiaries in the household. We managed this by excluding households that had members targeted by the strategy from the control group. Future ASRH strategies need to collect baseline data, as well as end of strategy data for full evaluation and for use by future studies. The study also does not focus on how the youth make ASRH decisions. There is need for further study to understand risk taking amongst the youth towards ASRH to inform the design of effective service delivery in the future.

## Conclusions

This study was undertaken with the expectation that the combined set of ASRH interventions coordinated at a national level in Zimbabwe would result in increased ASRH outcomes. Evaluating the effectiveness of this strategy, using the DID method combined with propensity score matching, the study concludes that the ASRH strategy resulted in improvements in HIV testing, STI treatment and reduced HIV prevalence trajectory. The study could not find evidence in support of any impact on condom use nor reduction of STI prevalence. Furthermore, the study did not find superior outcomes in regions with more resources. This suggests that the key for better outcomes from future ASRH strategies lies in redesigning service delivery approaches to target HIV hotspots and rural areas as well as improving the strategy’s coordination and monitoring, as well as aligning and enforcing government policies that promote sexual and reproductive health rights.

## Supporting information

S1 Table Impact of the ASRH strategy by gender. (DOCX)

S2 Table Impact of the ASRH strategy by household wealth status. (DOCX)

S3 Table Impact of the ASRH strategy by place of residence. (DOCX)
