# Supplementary material for: Impact of the adolescent and youth sexual and reproductive health strategy on service utilisation and health outcomes in Zimbabwe
Source: PLoS One. 2019 Jun 25;14(6):e0218588. doi: 10.1371/journal.pone.0218588 (PMC6592535; doi:10.1371/journal.pone.0218588)
Supplement: S1 Table — (DOCX) [file pone.0218588.s001.docx]

***S1 Table. Impact of the ASRH strategy by gender***

|  | (1) | (2) | (3) | (4) | (5) |
| --- | --- | --- | --- | --- | --- |
| VARIABLES | Condom use | STI Prevalence | STI Treatment | HIV Testing | HIV Prevalence |
| ***Panel (a) Males*** | | | | | |
| *Before* |  |  |  |  |  |
| Control | 0.792 | 0.024 | 0.574 | 0.405 | 0.098 |
| Treated | 0.740 | 0.004 | 0.306 | 0.116 | 0.033 |
| Diff (T-C) | -0.051 | -0.020** | -0.268* | -0.290*** | -0.066*** |
|  | (0.095) | (0.010) | (0.146) | (0.028) | (0.020) |
| *After* |  |  |  |  |  |
| Control | 0.753 | 0.029 | 0.303 | 0.625 | 0.095 |
| Treated | 0.765 | 0.025 | 0.494 | 0.653 | 0.030 |
| Diff (T-C) | 0.012 | -0.004 | 0.191 | 0.028 | -0.064* |
|  | (0.107) | (0.020) | (0.165) | (0.063) | (0.033) |
| *Diff-in-Diff* | 0.063 | 0.017 | 0.459** | 0.317*** | 0.001 |
|  | (0.143) | (0.022) | (0.220) | (0.069) | (0.039) |
| ***Panel (b) Females*** | | | | | |
| *Before* |  |  |  |  |  |
| Control | 0.398 | 0.034 | 0.538 | 0.727 | 0.227 |
| Treated | 0.436 | 0.002 | 0.221 | 0.253 | 0.042 |
| Diff (T-C) | 0.038 | - 0.032*** | -0.318*** | -0.474*** | -0.185*** |
|  | (0.229) | (0.012) | (0.092) | (0.031) | (0.031) |
| *After* |  |  |  |  |  |
| Control | 0.380 | 0.023 | 0.296 | 0.908 | 0.267 |
| Treated | 0.733 | 0.020 | 0.543 | 0.866 | 0.097 |
| Diff (T-C) | 0.354 | -0.003 | 0.247** | -0.042 | -0.170*** |
|  | (0.332) | (0.016) | (0.124) | (0.034) | (0.039) |
| *Diff-in-Diff* | 0.315 | 0.029 | 0.565*** | 0.432*** | 0.015 |
|  | (0.403) | (0.020) | (0.155) | (0.046) | (0.049) |

Standard errors in parentheses*** p<0.01, ** p<0.05, * p<0.1
