# Supplementary material for: Impact of the adolescent and youth sexual and reproductive health strategy on service utilisation and health outcomes in Zimbabwe
Source: PLoS One. 2019 Jun 25;14(6):e0218588. doi: 10.1371/journal.pone.0218588 (PMC6592535; doi:10.1371/journal.pone.0218588)
Supplement: S2 Table — (DOCX) [file pone.0218588.s002.docx]

***S2 Table. Impact of the ASRH strategy by household wealth status***

|  | (1) | (2) | (3) | (4) | (5) |
| --- | --- | --- | --- | --- | --- |
| VARIABLES | Condom use | STI Prevalence | STI Treatment | HIV Testing | HIV Prevalence |
| ***Panel (a) Rich*** | | | | | |
| *Before* |  |  |  |  |  |
| Control | 0.837 | 0.012 | 0.288 | 0.657 | 0.086 |
| Treated | 0.888 | 0.004 | 0.349 | 0.187 | 0.043 |
| Diff (T-C) | 0.051 | -0.008 | 0.060 | -0.470*** | -0.043** |
|  | (0.086) | (0.006) | (0.141) | (0.026) | (0.018) |
| *After* |  |  |  |  |  |
| Control | 0.866 | 0.024 | 0.513 | 0.818 | 0.176 |
| Treated | 0.800 | 0.022 | 0.582 | 0.768 | 0.064 |
| Diff (T-C) | -0.066 | -0.002 | 0.070 | -0.050 | -0.112*** |
|  | (0.114) | (0.017) | (0.181) | (0.047) | (0.039) |
| *Diff-in-Diff* | -0.117 | 0.006 | 0.009 | 0.421*** | -0.069 |
|  | (0.143) | (0.018) | (0.230) | (0.053) | (0.043) |
| ***Panel (b) Poor*** | | | | | |
| *Before* |  |  |  |  |  |
| Control | 0.736 | 0.038 | 0.627 | 0.447 | 0.265 |
| Treated | 0.480 | 0.004 | 0.285 | 0.182 | 0.031 |
| Diff (T-C) | -0.256 | -0.034** | -0.342*** | -0.265*** | -0.234*** |
|  | (0.156) | (0.015) | (0.116) | (0.043) | (0.040) |
| *After* |  |  |  |  |  |
| Control | 0.603 | 0.031 | 0.509 | 0.780 | 0.234 |
| Treated | 0.657 | 0.020 | 0.489 | 0.769 | 0.072 |
| Diff (T-C) | 0.054 | -0.011 | -0.020 | -0.011 | -0.162*** |
|  | (0.191) | (0.020) | (0.211) | (0.051) | (0.039) |
| *Diff-in-Diff* | 0.310 | 0.023 | 0.322 | 0.254*** | 0.072 |
|  | (0.246) | (0.025) | (0.241) | (0.067) | (0.056) |

Standard errors in parentheses

*** p<0.01, ** p<0.05, * p<0.1
