# Supplementary material for: Impact of the adolescent and youth sexual and reproductive health strategy on service utilisation and health outcomes in Zimbabwe
Source: PLoS One. 2019 Jun 25;14(6):e0218588. doi: 10.1371/journal.pone.0218588 (PMC6592535; doi:10.1371/journal.pone.0218588)
Supplement: S3 Table — (DOCX) [file pone.0218588.s003.docx]

***S3 Table. Impact of the ASRH strategy by place of residence***

|  | (1) | (2) | (3) | (4) | (5) |
| --- | --- | --- | --- | --- | --- |
| VARIABLES | Condom use | STI Prevalence | STI Treatment | HIV Testing | HIV Prevalence |
| ***Panel (a) Urban*** | | | | | |
| *Before* |  |  |  |  |  |
| Control | 0.886 | 0.015 | 0.444 | 0.655 | 0.086 |
| Treated | 0.864 | 0.003 | 0.236 | 0.173 | 0.049 |
| Diff (T-C) | -0.022 | -0.012 | -0.208 | -0.482*** | -0.037* |
|  | (0.097) | (0.008) | (0.155) | (0.029) | (0.020) |
| *After* |  |  |  |  |  |
| Control | 0.854 | 0.028 | 0.491 | 0.824 | 0.200 |
| Treated | 0.813 | 0.025 | 0.571 | 0.767 | 0.065 |
| Diff (T-C) | -0.042 | -0.003 | 0.080 | -0.058 | -0.135*** |
|  | (0.123) | (0.020) | (0.166) | (0.051) | (0.046) |
| *Diff-in-Diff* | -0.020 | 0.009 | 0.288 | 0.424*** | -0.098** |
|  | (0.156) | 0.022 | (0.227) | (0.058) | (0.050 |
| ***Panel (b) Rural*** | | | | | |
| *Before* |  |  |  |  |  |
| Control | 0.626 | 0.038 | 0.584 | 0.484 | 0.199 |
| Treated | 0.578 | 0.003 | 0.302 | 0.193 | 0.033 |
| Diff (T-C) | -0.048 | -0.035*** | -0.283*** | -0.291*** | -0.165*** |
|  | (0.125) | (0.011) | (0.093) | (0.030) | (0.026) |
| *After* |  |  |  |  |  |
| Control | 0.642 | 0.022 | 0.430 | 749 | 0.181 |
| Treated | 0.690 | 0.020 | 0.457 | 0.771 | 0.069 |
| Diff (T-C) | 0.049 | -0.002 | 0.027 | 0.022 | -0.112*** |
|  | (0.144) | (0.016) | (0.151) | (0.048) | (0.032) |
| *Diff-in-Diff* | 0.096 | 0.032* | 0.310* | 0.313*** | 0.053 |
|  | (0.190) | (0.019) | (0.178) | (0.056) | (0.041) |

Standard errors in parentheses

*** p<0.01, ** p<0.05, * p<0.1
